# Cat-bite-induced *Francisella tularensis* infection with a false-positive serological reaction for *Bartonella quintana*

**DOI:** 10.1099/jmmcr.0.005071

**Published:** 2017-02-28

**Authors:** Evelina Petersson, Simon Athlin

**Affiliations:** ^1^​ Department of Medicine, Karlskoga Hospital, Karlskoga, Sweden; ^2^​ Department of Infectious Diseases, Faculty of Medicine and Health, Örebro University, Örebro SE 701 82, Sweden

**Keywords:** tularaemia, *Francisella tularensis*, *Bartonella quintana*, serology, cross-reaction

## Abstract

**Introduction.** Tularaemia is caused by infection with *Francisella tularensis*transmitted via direct contact with an infected hare carcass or indirectly through the bites of vectors, but may be cat-bite-associated as well. Medical history and reliable diagnostic analysis are important in order to differentiate it from other cat-associated infections, e.g. *Bartonella* spp.

**Case** **presentation.** A healthy 56-year-old man was examined because of a cat-bite-associated ulceroglandular wound on his right thumb. Nineteen days after the cat bite occurred, a serology test was positive for anti-*Bartonella quintana*, but negative for anti-*F. tularensis*. Since *Bartonella* infections are rare in Sweden, another serology test was analysed 2 weeks later with a positive result for anti-*F. tularensis*. The patient was treated with doxycycline for 14 days and recovered. The patient was re-sampled after 18 months to obtain a convalescent sample. The acute and the convalescent samples were both analysed at a reference centre, with negative results for anti-*Bartonella* spp. this time.

**Conclusion.** This case is enlightening about the importance of extending the medical history and re-sampling the patient for antibody detection when the clinical suspicion of cat-bite-associated tularaemia is high. The false-positive result for anti-*B. quintana* antibodies may have been due to technical issues with the assay, cross-reactivity or both.

## Introduction

Tularaemia, a zoonosis caused by the small Gram-negative bacterium *Francisella tularensis,* is caused mainly via transmission from direct contact with an infected hare carcass or through the bites of vectors, such mosquitoes or ticks [1]. Infected cats are known to transmit the disease to humans [2, 3], and bites by infected squirrels and hamsters have also been reported to cause tularaemia [4]. The disease may also be contracted by inhalation or by ingestion of infected water or food. In Sweden, the majority of tularaemia cases occur during late summer and early autumn in the ulceroglandular form [5, 6]. A typical case would be a patient who developed a primary lesion after a mosquito bite on the leg, with high fever and engagement of the proximally located inguinal lymph nodes [2].

Here, we present a case of ulceroglandular tularaemia infection after a cat bite, with a false-positive reaction in the serological test for *Bartonella quintana* (the causative agent of trench fever). The case is enlightening about the importance of extending the medical history and re-sampling the patient for antibody detection when the clinical suspicion of tularaemia is high. The possibility of cross-reactivity with *B. quintana* is evaluated.

## Case Report

On January 14th 2015, a previously healthy 56-year-old man was examined by a general practitioner in Örebro, Sweden, due to the patient having a fever after a cat bite on his right thumb 19 days earlier. A small pustule had appeared on the thumb and had developed into a painless ulcerated lesion. During the preceding 10 days, he had experienced pain in his right axilla and a fever of 39.5 °C. The general practitioner suspected cat-scratch fever, caused by *Bartonella henselae*, and referred the patient to the Department of Infectious Diseases, Örebro University Hospital, Örebro, Sweden, for further examination.

At the hospital, the initial investigation revealed a rectal temperature of 38.2 °C and a heart rate of 100 beats min^−1^. The patient had an erythema of 12×15 mm with peeling skin on his right thumb, and an ulcer of 5×5 mm in the centre. He had a tender 20 mm lymph node in his right axilla. On his right upper arm, extending from the medial side of his elbow to his axilla, a mild lymphangitis was observed. No other abnormal findings were noted after examination of his lungs, skin and other regional lymph nodes. He had an elevated white blood cell count of 11.9×10^9^ cells l^−1^ (reference value 3.5–8.8×10^9^ cells l^−1^), a C-reactive protein level of 23 mg l^−1^ (reference value <4 mg l^−1^), and elevated liver transaminaselevels – an alanine transaminase level of 2.2 µkat l^−1^ (reference value <1.1 µkat l^−1^) and an aspartate transaminase level of 1.01 µkat l^−1^ (reference value 0.20–0.80 µkat l^−1^). A volume of 8–10 ml of venous blood was inoculated in each of two BACTEC Plus Aerobic/F bottles and two BACTEC Plus Anaerobic/F bottles, with no growth of bacteria after 8 days. A wound culture from the right thumb showed only coagulase-negative staphylococci, which was considered as non-significant.

The patient and his wife, as well as the cat, had no history of louse-contact previously and lived in a modern apartment with good conditions. He denied any contact with other animals or exposure to animal droppings. On suspicion of cat-scratch fever or tularaemia, serum samples were taken 19 days after the cat bite occurred and analysed for anti-*Bartonella* spp. antibodies and anti-*F. tularensis* antibodies. Treatment was then initiated with doxycycline, 200 mg once a day for 2 weeks. At the follow-up contact, by telephone after 7 days, the patient was completely recovered. The laboratory's request form for anti-*Bartonella* spp. immunoglobulins included testing for both anti-*B. henselae* antibodies and anti-*B. quintana* antibodies. The assays were performed by indirect immunofluorescence test (Euroimmun kit) for IgG and showed a negative result for anti-*B. henselae* (IgG titre <1 : 64), but a positive result for anti-*B. quintana* (IgG titre 1 : 128). Briefly, the assays were performed manually by incubation of human serum on a glass slide together with fluorescent secondary antibodies and the results were evaluated by fluorescence microscopy. The serological analysis for anti-*F. tularensis* antibodies, performed with a tube agglutination test (Widal reaction), was negative (IgM/IgG titre <1 : 64).

Since *B. quintana* infections are rare in Sweden, the diagnosis was considered unlikely and the patient was contacted again by telephone. He explained that the cat was brought to his recreational (holiday) cottage 2 weeks before the cat bite occurred, and a dead hare had been found in the cottage garden where the cat had its territory. Since the time of exposure, the cat had not shown any signs of disease; it was later examined by a veterinarian, but had no signs of tularaemia nor louse infection. On suspicion of ulceroglandular tularaemia indirectly transmitted by the cat's saliva, a second serum sample for anti-*F. tularensis* antibodies was taken 32 days after the cat bite occurred. This time the assay was positive (IgG titre 1 : 320) and a final diagnosis of tularaemia was made. The positive serological test for anti-*B. quintana* antibodies was considered as a false-positive result.

Since *B. quintana* may be a chronic infection and antibodies may persist over many years [7], we resampled the patient 18 months after the cat bite occurred, and analysed the frozen acute (2015) sample along with the convalescent (2016) sample. This time, the paired samples were negative for anti-*B. henselae* (IgG titres <1 : 64 and <1 : 64, respectively) and anti-*B. quintana* (IgG titres 1 : 64 and <1 : 64, respectively) antibodies. We also sent the acute and convalescent samples to a reference laboratory at the Centre National de Référence sur les Rickettsioses et la Tularémie, Aix Marseille Université, France, for evaluation. The paired samples were again negative for anti-*B. henselae* (IgG titres <1 : 100 and <1 : 100, respectively) and anti-*B. quintana* (IgG titres <1 : 100 and <1 : 100, respectively) antibodies by using a microimmunofluorescence assay, as described elsewhere [8, 9].

## Discussion

Tularaemia is associated with a variety of clinical manifestations similar to those seen in other vector-borne infections [10]. Cat-associated tularaemia is uncommon, but has been reported before in Sweden [6] and other countries [11]. To distinguish tularaemia from other infections may be difficult and requires a detailed medical history from the patient, and sensitive and specific laboratory methods, as well as a re-sampling of the the patient when the diagnosis is unclear. *F. tularensis* cultivation should be avoided due to the risk of laboratory transmission [12] and as the bacterium does not grow in routinely plated cultures [13]. PCR applied on samples from primary lesions is a fast and sensitive method [12]. The most common method used for diagnosing tularaemia is by detection of antibodies in serum by agglutination or enzyme-linked immunosorbent assays [13, 14].

Diagnosing tularaemia by antibody detection has several limitations. Firstly, IgM and IgG appear together, but are often not detectable until 2 to 3 weeks after infection [15, 16]. If the first serology is analysed too early, a negative result may be fallacious and the patient should be resampled after a few weeks. Secondly, cross-reactions have been described between *F. tularensis* and species of *Brucella*, *Proteus* and *Yersinia* [12, 16]. Moreover, false-positive and false-negative serological test results may occur due to technical problems with the assays or inadequate performance of an assay by the technician.

In this report, the positive titre for anti-*B. quintana* antibodies (1 : 128) was unexpected, since clinical infections with the bacteria are uncommon [17, 18] and it has not been associated with cat bites previously in Sweden [19, 20]. Infections caused by *B. quintana* are exclusively seen in patients with actual or recent contact with body lice [21]. In this case, neither the patient nor the cat had been exposed to lice. Also, in Sweden the seroprevalence of anti-*B. quintana* antibodies is very low among humans [22, 23] and cats [24].

*B. quintana* has been reported to cross-react within the genus *Bartonella* [25, 26], and with species of *Chlamydophila* [27, 28] and *Coxiella* [9]. In clinical samples, cross-reactivity between *F. tularensis* and *B. quintana* has not been described to our knowledge. However, it is known that *F. tularensis* and *Bartonella* spp. express common proteins that may elicit an antibody response in infected individuals [29]. In a previous study by Gilmore *et al.* [30], rabbit anti-*F. tularensis* antibodies reacted with a recombinant immunogenic protein (SucB) derived from *B. quintana*. In another study, Litwin *et al.* [31] showed that a recombinant SucB protein from *B. henselae* cross-reacted with human serum containing anti-*F. tularensis* antibodies. The *B. henselae sucB* gene used in the study was determined to have 85.3 % identity to the SucB protein of *B. quintana.* These findings indicate that cross-reactions between *F. tularensis* and *B. quintana* are possible in immunological assays on clinical samples.

In this report, a significant titre of anti-*B. quintana* antibodies was detected by the Euroimmun assay in the initial serum sample, but not when the sample was re-analysed, and it was negative by the microimmunofluorescence assay. This may be explained by technical challenges in the performance of the Euroimmun assay and/or by differences in the specificities of the two assays. A limitation of this study was that we did not evaluate the two assays with multiple samples from *F. tularensis-*positive patients and we did not re-evaluate the serum samples after neutralization of anti-*F. tularensis* antibodies to evaluate positive results for anti-*B. quintana* antibodies. Therefore, we suggest that future evaluations of immunoassays for anti-*F. tularensis* antibodies should include specificity analysis of cross-reactivity with anti-*B. quintana* antibodies and that clinicians should be aware of potentially false-positive results in tularaemia patients.
